# Evolution of Artificial Hearts: An Overview and History

**DOI:** 10.14740/cr354w

**Published:** 2014-10-06

**Authors:** Sanna Khan, Waqas Jehangir

**Affiliations:** a Drexel University College of Medicine, Philadelphia, PA, USA; b Raritan Bay Medical Center, Perth Amboy, NJ, USA

**Keywords:** Heart, Artificial Heart, Evolution

## Abstract

The heart is a muscular organ which pumps blood through blood vessels to different organs of the body. It is the most significant and vital organ in the human body. Without this organ, life is unimaginable. Doctors and scientists have been trying for a long time to create something similar or equivalent to the heart. The purpose is to develop a temporary machine or pump for a person who has a disease of the heart and their survival without transplant is impossible. These temporary devices can provide enough time for the patient until a donor heart is available. The purpose of this review is to provide an overview and history of how man has developed an artificial heart for survival.

## Introduction

The heart is the most important organ in the human body. It transports blood to various parts of our body and most importantly keeps us alive. The leading cause of death today is heart disease and around 600,000 people die from this yearly [1]. Furthermore, heart failure is affecting around 20 million people exclusively in the United States and Europe [2]. Approximately 100,000 people are diagnosed with progressive heart failure every year [2]. If the heart failure has reached an advanced stage, “60-94%” of patients will die in 1 year [2]. Due to these statistics, the heart has become a concern for doctors and scientists alike. There are many drugs in the market to treat heart disease but once the disease has reached a certain phase, the drugs can only do so much. The only option left after drugs is transplantation. Patients are then left to wait on a donor list but some will not make it waiting to receive a transplant. Additionally, there are approximately a little over 2,000 hearts available for patients per year but there are more than 3,000 patients waiting for a transplant [2, 3]. Throughout the last century, many doctors have materialized their theories concerning the heart into new life-saving technologies. These theories culminated over the years into what is known as the artificial heart today.

## Review of Literature and Discussion

The theory of “mechanical circulatory support” was first hypothesized by Julien Jean Cesar LeGallois in 1812 [4]. However, his hypothesis would not become a reality until the 21st century. Nearly a century after LeGallois’ theory, the unforeseen pairing of Charles Lindbergh and Alexis Carrel joined forces to advance mechanical circulatory support in the 1920s. Lindbergh was an inventor who is mainly known today as an aviator who flew over the Atlantic whilst Carrel was a surgeon who won the Nobel Prize for his innovations in organ transplantation [5]. Carrel was uncertain that while performing heart surgery, an external blood pump could support the human body. Lindbergh reviewed the issues that Carrel was having and went on to create several of his own blood pumps which turned out to be unsuccessful. It took him a few years but he eventually created a blood pump that actually worked. Lindbergh also went on to create a centrifuge that could securely separate blood plasma. After this innovation, Carrel and Lindbergh went on to create an “*in vitro* artificial heart-like” device to keep organs alive when removed from the body [6]. Some organs they removed were kidneys, hearts, thyroid glands, and ovaries. These organs were monitored after removal to observe their development and function [6]. Early innovations such as an external blood pump and device similar to that of an artificial heart set into motion the desire to create a total artificial heart.

Subsequently in 1937, Dr. Vladimir P. Demikhov developed a total artificial heart (TAH) device and executed the first coronary artery bypass surgery and intrathoracic transplantation in the world [7, 8]. The TAH he developed was made up of two pumps, besides each other which were driven by an “external motor with a transcutaneous drive shaft” [8]. This device was transplanted into a dog that went onto live 5.5 h after the operation. His experiments were the first of this kind and he mainly tested on dogs. In 1946 Dr. Demikhov simultaneously transplanted a heart and lung, which proved successful. He was able to do these operations without using a cardiopulmonary bypass. In lieu of the cardiopulmonary bypass, Dr. Demikhov performed surgery quickly and used his own method to preserve organs during transplantation. In June 1946, he performed a heterotopic heart-lung transplant on a dog for 9.5 h, which was a landmark in his experiments. The animals Dr. Demikhov experimented on usually survived 30 days post operation. Dr. Demikhov’s donor’s heart-lung preparations were preserved during transplantation by utilizing “closed-circuit circulation” [7]. “Blood from the left ventricle was pumped into the aorta; then, through the coronary vessels that supplied the myocardium, it passed into the right atrium, the right ventricle, and the lungs, where the blood was reoxygenated and returned to the left atrium” [7]. In July 1953, he reached a milestone by performing the first successful coronary bypass surgery on a dog. The International Society for Heart and Lung Transplantation awarded the “first Pioneer Award” to Dr. Demikhov in 1989 for “the development of intrathoracic transplantation and the use of artificial hearts” [7]. In summation, Dr. Demikhov was a pioneer who developed his ideas about the heart into reality which are now regularly used in medicine.

A few years after Dr. Demikhov in 1939, Dr. John H. Gibbon, Jr. of the United States was assisting another physician in an emergency pulmonary embolectomy where a patient lost consciousness due to blood clots being removed in open-heart surgery. He thought that if there was some device which can eliminate blood from the patient’s body while avoiding the lungs, oxygenate the body, and then return to the heart, the patient would still be alive. This event fueled his passion to create the heart-lung machine [9, 10]. Over the next several years, Dr. Gibbon went on to develop an apparatus which was studied on laboratory rats. The lab rats survived total body perfusion experiments and this research went on to be published in 1939. Years later, Dr. Gibbon went on to work for Jefferson Medical College where he teamed up with IBM to work on his device which after development was introduced as IBM Model I. This device was successful when used on dogs; however, the use was limited on humans. In 1952, the Model II heart-lung device was released to be used on humans. Though the machine was well-designed, a baby just over a year old, the first patient, died while the surgery was taking place. In 1953, the device was used on two more children who also died. After this, Dr. Gibbon halted all work with the device. In July 1954, after further research on clots and blood loss, the device was revamped and Model III was released by IBM [9, 10]. However, Dr. Gibbon already shared his device with the Mayo Clinic in 1953 and they went on to advance the device, naming it the “Mayo Gibbon-type oxygenator” which went on to save hundreds of patients [9, 10].

In 1948, William H. Sewell, Jr., a medical student at Yale University sought after building an artificial heart for his thesis to graduate from medical school. Dr. Sewell had seen Dr. Gibbon and others make advancements in cardiology. Dr. Gibbon had basically created a heart-lung machine to function in place of the heart and lungs during heart operations; however, when Dr. Sewell began his research, no heart operations using a pump-oxygenator had been produced [11]. He thought that the patient’s lungs could be used to oxygenate the blood during a heart operation and as a result only a single or a pair of pumps would be required in place of the heart. Dr. Sewell designed his pump so that it would go around the right side of the heart [11]. In his early trials, he was not successful. His experiments failed because he could not push blood through rubber tubing using a roller pump. He then decided to use pressurized gas to create force. Dr. Sewell also studied the methods which different researchers had used but they also did not move blood through a pumping chamber [11]. Dr. Sewell then designed a “pneumatically-powered pump” [11]. This pump was made up of “tubular glass pumping chamber with a side arm connected to a compressed air and vacuum source and a rubber bladder made of reinforced Penrose tubing held in place by perforated rubber stoppers” [11]. The pumping chamber was protected by rubber-flap valves and suctioned blood from the right atrium into the pulmonary artery via a cannula. Eccentric cams and weighted arms with blades controlled the timing of compressed air and suction since the blades stopped and released the small rubber tubes which all resulted in compressed air and vacuum sources [11]. This method proved successful for Dr. Sewell’s pump and he went on to win the thesis prize from his medical school for this achievement.

Following in the footsteps of their predecessors, Dr. Tetsuzo Akutsu and Dr. Willem Kolff from the Cleveland Clinic in the United States were the first to successfully implant a TAH in an animal that went on to live for 1.5 h in 1957. Both of these doctors were experts in developing TAHs and received many accolades [12]. A few years later in 1952, Domingo Liotta of Argentina created his own models similar to that of Dr. Tetsuzo and Dr. Kolff. His models increased survival time up to 13 h [8]. Liotta then went to work with Dr. Michael DeBakey of Baylor University in the United States, who in 1983 went on to develop a device using a roller pump which could transfuse blood constantly [4]. In 1963, Liotta carried out the “first clinical implantation of a pulsatile left ventricular assist device” [8]. A few years later in 1969, Dr. Denton Cooley executed the “first TAH” transplantation in a human [8]. Dr. Cooley was also working with Domingo Liotta. They were working together to advance the artificial heart that Liotta had initially created [13]. Silastic containing Dacron made the stretchy barrier of the heart [13]. A net-like texture was used to imitate vascular grafts. Wada-Cutter hingeless valves were utilized because of their wide opening which allowed an easy flow. There were some issues with these valves that turned out to be beneficial because the valves caused a disgorging that deterred thrombus formation. Ironically, thrombus formation was an issue in all TAHs that came out after this. Dr. Cooley and Liotta then sought the advice of an engineer to create a “pneumatic drive console” [13]. A “pneumatic drive console” allowed their new design of the artificial heart to be used in humans [13]. Thereafter, this device was implanted into a 47-year-old male who was nearly incapacitated and had a history of having heart attacks for 10 years. This patient received this device, the first TAH in 1969 and it seemed fine at first; however, his renal function started to decline and hemolysis started [13]. A donor had to be located for a human heart transplant 64 h after the initial surgery and patient went on to die 32 h after the human heart transplant due to pneumonia. The patient mainly died due to anti-rejection. Though the patient died, Dr. Cooley learned that human circulation by a mechanical device had the potential to be successful [13].

In the timeline of events of the advancement of the artificial heart, the next individuals have been the focus of a controversy. Paul Winchell claims that he was the first to have invented the artificial heart and that Dr. Robert Jarvik copied his ideas to invent the Jarvik heart. However, upon research it was found that he was not the first because prior patents had been filed before Winchell’s [14].

Dr. Robert Jarvik is renowned for his work on the first permanent artificial heart which proved to be successful. He has received the most acclaim amongst all of the previous inventors. In 1982, the first permanent artificial heart was transplanted into a 61-year-old patient named Barney Clark by surgeons at the University of Utah. Dr. Willem Kolff, who was mentioned earlier, led the team that worked on this artificial heart. After leaving the Cleveland Clinic in 1967, Dr. Kolff went on to join the University of Utah. It is there in 1971 where he met and hired Robert Jarvik onto his study steam, which was working to develop artificial organs. Dr. Kolff had a tradition of naming the artificial hearts after the investigators who were working on them. Robert Jarvik happened to be working on the artificial heart and therefore it came to be known as the Jarvik 7. Dr. Jarvik was only 35 years old when we became famous for and received all credit for this invention simply because it was named after him and Dr. Kolff has been forgotten [15]. Before its first successful transplantation in 1982, the Jarvik 7 was tested in clinical trials. Barney Clark, the first recipient of the Jarvik 7 lived for 112 days after the transplant. The second recipient went on to live for 620 days. In the three subsequent recipients, one died from blood loss, and the other two lived for 10 and 14 months [16]. Essentially, all patients died from different complications such as multi-organ failure, stroke, and infection to name a few. The main issue with the Jarvik 7 was that a “large pneumatic console” was required for treatment and therefore the patient could not leave the hospital [16]. This caveat would not allow the Jarvik 7 to be a permanent artificial heart implant.

Essentially, the Jarvik 7 had two “air-powered pumps” that copied the heart’s function at 40 - 120 bpm (beats per minute) [16]. Each chamber had a “disk-like mechanism” which was made out of polyurethane that pushed the blood through the Jarvik 7 from the inflowing valve to the exiting valve [16]. Cuffs were used to attach the Jarvik 7 to the heart’s natural atria. The cuffs were attached by drive lines which were made of reinforced polyurethane. The drives lines were also coated to promote tissue growth. The drive lines were inserted through the patient’s left side. A large electronic unit the size of a refrigerator supplied power to the Jarvik to allow it to operate. This unit also controlled the “pump rate, pumping pressure, and other essential functions using electricity, compressed air, and a vacuum” [16]. Jarvik 7 was later renamed the Cardiowest Total Artificial Heart. This is because Symbion, who originally produced the device, haltered manufacturing in 1990 because they were not following FDA requirements. MedForte Research gained rights from Symbion and it later formed a partnership with University Medical Center in Tucson, Arizona [17]. These two organizations went on to form the CardioWest heart. Consequently, the Jarvik 7 was then renamed to the Cardiowest Total Artificial Heart in 1991 [16, 17]. In 2004, the Cardiowest TAH received FDA approval for bridge to transplant indications [2]. Bridge to transplant basically means that the artificial heart is only in place till an actual human heart can be located for transplantation. Several years later, the CardioWest TAH was again renamed “the SynCardia temporary” TAH in 2010. Clinical trials showed survival rates to be “79% vs. 46%” when evaluated against the control group [2, 18]. The survival rates 1 and 5 years after transplantation were “86% and 64%” [2, 18]. These statistics proved to be positive when compared with the statistics of that of the United Network of Organ Sharing [2].

After the SynCardia temporary, the AbioCor TAH emerged. This is the first “self-contained internal artificial heart” [19]. Researchers studied and tested this device for 30 years. Clinical trials began in 2001 and that same year the device was implanted in a human [19]. The AbioCor went on to be approved by the FDA in 2006 [19]. What is unique about this device is that it does not require any subcutaneous connections, meaning that that patient does not need to be hooked up to “external air-pumping machines via tubes or wires that pierce the skin’s surface” [20]. The AbioCor TAH weighs 2 pounds and is constituted of four parts which are implanted into the human body. These four parts are: electronic controller, thoracic unit, lithium battery, and transcutaneous energy transmission device (TET) [20]. This also includes “two artificial ventricles and corresponding valves”. This system has a hydraulic pump which is run by a motor and mimics the human heartbeat. The battery that is implanted into the human body is continuously recharged by the TET and an external battery. The TET relays energy via the skin. The internal battery has power for up to 30 min whereas the external battery can last up to 4 h. It should be noted the AbioCor system is only geared towards patients who have biventricular heart failure [20]. Biventricular heart failure occurs when both the left and right ventricles are not pumping a sufficient amount of blood to maintain the body [21]. Basically, the FDA had granted that the AbioCor only be used under the Humanitarian Device Exception (HDE) [22]. Following this exception, only one patient was transplanted with an AbioCor and it later went out of production due to complications from “thromboembolism and atrial suck-down events” [22].

In March 2010, SynCardia released the portable freedom driver [23]. With this device, patients will no longer be confined to a hospital due to the large pneumatic console. This will allow patients freedom and the ability to live their lives normally after being transplanted with an artificial heart. After receiving an artificial heart, patients are normally restricted to the hospital waiting for a human heart donor. This reduces the quality of life and incurs costs for the patients. Additionally, hospitals do not have the resources to maintain the current protocol. The portable freedom driver weighs 13 pounds and is basically a “piston-driven pneumatic compressor” that supplies pressure to the TAH. The beat rate is the only parameter which is adjustable and is calculated prior to being connected to the patient [24]. The purpose of the beat rate is to partly fill the ventricles. Thus, the TAH output produces a Frank Starling effect. A Frank Starling effect is when “the stroke volume” of the heart rises because blood has filled the heart [25]. The extra amount of blood causes the ventricular wall to expand, which in turn triggers the cardiac muscle to contract vigorously [25]. After the Frank Starling response, electric motors within the device push the piston to allow backup superfluity [24]. The patient can easily charge the portable freedom driver in any electrical outlet even via a car’s auxiliary port. Patients even have the ability to bath with this device. The device contains lithium batteries which last for 3 h [24]. This unique device allows the recipients of the TAH to be outpatients during their wait for a human heart donor. They would no longer be restricted to the hospital.

It should be noted that though the artificial heart is an amazing accomplishment which has been made, it is also extremely costly. It can cost anywhere from $190,000 to $220,000 [26]. More than 600 patients have been transplanted with the SynCardia TAH [18]. This quantity is greatly significant when compared to the nearly 15 patients who received the AbioCor TAH [18, 22]. The SynCardia is the most used TAH, making up 93% of the worldwide use of this device [18]. It is the most successful TAH and exceeds “116 patient-years of device support” [18].

## Conclusion

The heart is a vital organ needed for human survival. It supplies oxygen to the major organs of our body. If the heart does not function properly, different organs such as the liver, kidneys, and brain will not receive oxygen. This would cause multi-organ failure and brain death. Currently, heart disease is the primary cause of death in the United States [1]. Doctors and engineers over the last century have made significant advances to create the artificial heart. The current purpose of an artificial heart is not to replace the actual human heart, but rather it is a temporary placement to sustain life till a human heart can be located for transplant. Up till now, no artificial heart created has been without fault. Most recipients suffer from infection. Given the advancements made over the last century, doctors may be able to completely sustain life through an artificial heart, since they are improving each time.
